# Disruption of *Phthorimaea operculella* (Lepidoptera: Gelechiidae) oviposition by the application of host plant volatiles

**DOI:** 10.1002/ps.3597

**Published:** 2014-08-09

**Authors:** Gianfranco Anfora, Silvia Vitagliano, Mattias C Larsson, Peter Witzgall, Marco Tasin, Giacinto S Germinara, Antonio De Cristofaro

**Affiliations:** a Research and Innovation Centre, Fondazione Edmund Mach, San Michele all'AdigeTrentino, Italy; b Department of Agricultural, Environmental and Food Sciences, University of MoliseCampobasso, Italy; c Faculty of Science and Technology, Free University of BolzanoBolzano, Italy; d Chemical Ecology, Department of Plant Protection Biology, Swedish University of Agricultural SciencesAlnarp, Sweden; e Department of the Sciences of Agriculture, Food and Environment, University of FoggiaFoggia, Italy

**Keywords:** potato tuberworm, semiochemicals, single-cell recording, behavioural bioassays, egg-laying disruption

## Abstract

**BACKGROUND:**

***Phthorimaea operculella* is a key pest of potato. The authors characterised the *P. operculella* olfactory system, selected the most bioactive host plant volatiles and evaluated their potential application in pest management. The electrophysiological responses of olfactory receptor neurons (ORNs) housed in long sensilla trichodea of *P. operculella* to plant volatiles and the two main sex pheromone components were evaluated by the single-cell recording (SCR) technique. The four most SCR-active volatiles were tested in a laboratory oviposition bioassay and under storage warehouse conditions**.

**RESULTS:**

**The sensitivity of sensilla trichodea to short-chained aldehydes and alcohols and the existence of ORNs tuned to pheromones in females were characterised. Male recordings revealed at least two types of ORN, each of which typically responded to one of the two pheromone components. Hexanal, octanal, nonanal and 1-octen-3-ol significantly disrupted the egg-laying behaviour in a dose-dependent manner. Octanal reduced the *P. operculella* infestation rate when used under storage conditions**.

**CONCLUSIONS:**

**This work provides new information on the perception of plant volatiles and sex pheromones by *P. operculella*. Laboratory and warehouse experiments show that the use of hexanal, octanal, nonanal and 1-octen-3-ol as host recognition disruptants and/or oviposition deterrents for *P. operculella* control appears to be a promising strategy. © 2013 The Authors. Pest Management Science published by John Wiley & Sons Ltd on behalf of Society of Chemical Industry**.

## 1. INTRODUCTION

*Phthorimaea operculella* (Zeller) (Lepidoptera: Gelechiidae), the potato tuberworm, is a key pest causing severe damage to potato, which, in turn, is a food crop of essential importance worldwide. The pest is indigenous to South America but at present is spreading in all the potato production areas in temperate and subtropical climatic regions.^1^ Gravid females can lay eggs on foliage and soil next to the host plant or directly near the eye buds of tubers exposed through soil cracks or when they are kept under storage. Larvae develop endophytically in leaves, stems and tubers and feed on different Solanaceae, with a preference for potato (*Solanum tuberosum* L.).^1^ In addition to direct damage, galleries inside tubers facilitate the entrance of pathogens responsible for further severe losses, which may reach up to 100% under inadequate storage conditions, as often happens in developing countries.^1^

Until now, control of this pest has been carried out mainly using chemical insecticides, which are harmful to beneficial insects and cause pesticide resistance and environmental concerns.^2^,^3^ As for semiochemicals, the female sex pheromone of *P. operculella* has been identified as a mixture of (*E*,*Z*,*Z*)-4,7,10-tridecatrienil acetate [(*E*,*Z*,*Z*)-4,7,10-13:Ac] and (*E*,*Z*)-4,7-tridecadienil acetate [(*E*,*Z*)-4,7-13:Ac].^4^–^6^ Sex pheromone has been successfully employed in integrated pest management programmes using traps for pest monitoring, whereas pheromone-based control techniques, such as mass trapping, attract-and-kill and mating disruption, are feasible^7^–^9^ but not always satisfactory and not yet commercially viable. In spite of the great economic importance of *P. operculella*, little attention has been paid to the possible use of plant volatiles in control methods. Behavioural studies using plant material indicated that host plant recognition and selection by gravid females are mediated by chemical cues,^10^–^12^ while roughness of tuber surface is considered to be an important factor affecting the subsequent choice of the oviposition site.^13^,^14^ Two electroantennographic (EAG) studies showed the capability of the peripheral olfactory system of potato tuberworm adults to perceive a broad range of volatiles identified from potato leaves and tubers.^11^,^15^ High EAG sensitivity to green-leaf volatiles, particularly aldehydes and alcohols, has been found, although there are some apparent inconsistencies in the rank order of EAG responses to either single compounds or classes of chemicals.^11^,^15^

Long sensilla trichodea are the most abundant olfactory structures on the surface of both male and female antennae of the potato tuberworm^16^ and the pink bollworm,^17^ another important pest of the Gelechiidae family. However, no studies aimed at characterising male and female olfactory receptor neurons (ORNs) housed in the antennal sensilla trichodea of potato tuberworm have been carried out.

Hence, the goal of this study was to increase knowledge of the sensitivity of the *P. operculella* peripheral olfactory system to some electrophysiologically active host plant volatiles, previously identified, by single-cell recording (SCR) from sensilla trichodea ORNs of both sexes. Moreover, the effects of increasing concentrations of the most SCR-active compounds on the egg-laying behaviour of gravid females were assessed using a no-choice experimental design. Finally, the potential of one of the most bioactive compounds to disrupt potato tuberworm oviposition was evaluated in a traditional warehouse for potato storage.

## 2. MATERIALS AND METHODS

### 2.1 Rearing

Developmental stages of *P. operculella* were field collected in the Molise Region (Central Italy) and reared in the laboratory on tubers of the Spunta and Desireé susceptible varieties cultivated organically. Rearing cages were kept in a climatic chamber at 26 ± 2 °C and 60 ± 5% relative hunidity (RH) with a 16:8 h L:D photoperiod at 2000 lux light intensity. Every 6 months, new wild insects were introduced into the laboratory colony.

### 2.2. Odour stimuli

Compounds tested in electrophysiological assays are listed in Table1. Chemicals were purchased from Sigma-Aldrich (Milan, Italy) and were 85–99% pure. They were selected from compounds identified in the headspace of potato tubers and leaves and were chosen to represent the most EAG-active compounds in different chemical categories and the differential sensitivity between sexes.^11^,^15^ Furthermore, one plant ketone and acetate and two sex pheromone components of potato tuberworm were tested in SCR experiments. For each compound, 100 µg µL^−1^ stock solutions in both light mineral oil and hexane (Sigma-Aldrich, Milan, Italy) were prepared and stored at −20 °C.

**Table 1:** Activation of receptor neurons housed in long sensilla trichodea on the antennae of female *Phthorimaea operculella* by 13 host plant volatiles and the two main pheromone components^a^

Stimuli	Recordings^b^																			Number^c^
	1	2	3	4	5	6	7	8	9	10	11	12	13	14	15	16	17	18	19	
*Aldehydes*																				
Hexanal	**	***	***	**	**	0	**	0	0	0	***	***	**	***	***	0	**	0	***	13 (27)
Heptanal	0	0	^*^	**	0	***	**	0	0	0	0	**	***	0	0	0	0	0	^*^	7 (27)
Octanal	***	0	***	***	***	0	0	0	0	0	0	^*^	***	^*^	^*^	0	^*^	0	***	10 (27)
Nonanal	0	**	**	^*^	0	***	^*^	0	0	0	0	^*^	***	0	0	0	^*^	0	^*^	9 (27)
(*E*,*E*)-2,4-Decadienal	0	**	***	^*^	0	***	^*^	0	0	0	0	**	***	0	0	^*^	0	0	***	9 (27)
*Alcohols*																				
2-Hexanol	***	0	**	^*^	**	0	0	***	***	0	**	0	***	0	0	0	0	*	0	8 (27)
1-Pentanol	—	—	—	—	—	—	—	—	—	—	0	0	***	0	0	0	0	0	0	1 (16)
1-Octen-3-ol	***	***	*	0	0	***	**	***	0	*	***	**	***	**	0	0	0	0	**	12 (27)
*Acids*																				
Hexanoic acid	—	—	**	**	0	*	**	0	0	0	0	0	***	**	0	0	0	0	**	7 (25)
*Ketones*																				
2-Decanone	—	—	—	—	—	—	—	—	—	—	**	0	0	0	*	*	0	0	0	3 (16)
*Acetates*																				
Undecyl acetate	—	—	—	—	—	—	—	—	—	—	0	*	**	0	0	0	0	0	0	2 (16)
*Monoterpenes*																				
(*S*)-(−)-Limonene	—	—	—	—	—	—	—	—	—	—	0	0	0	0	0	0	0	0	0	0 (16)
*Sesquiterpenes*																				
(−)-Linalool	—	—	—	—	—	—	—	—	—	—	0	**	***	**	0	0	0	0	*	4 (16)
*Sex pheromones*																				
(*E*,*Z*,*Z*)-4,7,10-13:Ac	—	**	***	**	0	***	**	0	0	0	0	0	0	0	0	0	0	0	0	5 (26)
(*E*,*Z*)-4,7-13:Ac	—	—	—	—	—	***	**	0	0	0	0	0	**	0	0	0	0	0	0	3 (22)

a Each number in the table represents one single-cell recording. Eight recordings in which neither of the receptor neurons responded to any of the stimuli were not included in the table.

b —: compound not tested; 0: no response by any receptor neuron

* weak response, spike frequency increase with 10–40 Hz after stimulation

** intermediate response, spike frequency increase with 40–70 Hz

*** strong response, spike frequency increase with 70 Hz or more.

c The number of recordings in which a compound elicited responses of receptor neurons in the total of recordings (in brackets).

In SCR experiments, a 10 µL aliquot of 1 µg µL^−1^ hexane solution of a test compound was adsorbed on a piece of filter paper (1 cm^2^), exposed to the air for 1 min to allow solvent evaporation and inserted into a Pasteur pipette.

In oviposition bioassays, stimuli were 100 µg µL^−1^ mineral oil solutions of hexanal, octanal, nonanal and 1-octen-3-ol loaded into 35 mL volume polyethylene vials, with a 1 mm diameter hole in the lid, at doses of 4, 40 and 400 mg. In the warehouse experiment, a 1:1 (v/v) mineral oil solution of octanal loaded into low-void polymer disc dispensers (Trécé Inc., Adair, OK) (10 mL dispenser^−1^) was used.

### 2.3 Single-cell recording

Insects (2–3 days old) were placed in plastic pipette tips, with the head and antennae protruding from the cut-off tip. The specimen was placed onto a glass side, and the antennae were fixed on a cover glass with double-sided sticky tape. Dental wax (Surgident; Heraeus Kulzer Inc., South Bend, IN) was used further to restrain the head and keep the antennae from moving.

An electrolytically sharpened (in a 10% KNO_3_ solution) tungsten wire was used to penetrate the insect head cuticle to serve as a ground electrode. Another electrolytically sharpened tungsten electrode was inserted at the base of the sensilla to establish contact with the receptor neurons.^18^ The recording electrode was positioned by means of a DC-3K micromanipulator with a piezo translator (Märzhauser, Wetzlar-Steindorf, Germany) under a high-powered compound microscope with up to 500× magnification. Recordings were made from the curved hair-like olfactory sensilla trichodea, the most abundant on the surface of both male and female potato tuberworm antennae,^16^,^17^ selecting a recording area between the twentieth and thirtieth antennomere. During the recording, the antennae were continuously flushed with charcoal-filtered and humidified air at a rate of 20 mL s^−1^ through a glass tube, the outlet of which was about 1 cm from the preparation. A Pasteur pipette loaded with the odour stimulus was inserted into a hole in the glass tube, 15 cm from the outlet. A stimulus controller (Syntech, Hilversum, The Netherlands) connected to the pipette generated an air puff of 3 mL s^−1^ during 0.5 s through the pipette into the constant air flow over the antenna. The stimulations were presented with an interval of at least 30 s, or until the activity of ORNs had returned to their resting frequencies. Pipettes were renewed after five stimulations. Only recordings showing the activity of at least one sensitive neuron not responding to the control stimuli (a blank pipette and a pipette loaded with hexane) were considered in the data analysis.

Neuronal activity was monitored by computer loudspeakers, and the amplified analogue signal was captured and processed with a data acquisition controller (IDAC-4; Syntech). Spike frequencies of amplified signals were analysed using Autospike 3.2 software (Syntech). The response from receptor neurons was calculated as the difference between the number of spikes during 1 s before stimulus application and the number of spikes 1 s upon stimulation. Responses elicited in the ORNs were assigned to three different categories, according to different levels of increase in spike frequency (see Table1).

### 2.4 Oviposition bioassay

Bioassays were carried out in plastic cylindrical cages (20 × 12 cm ID) with the opening covered by a plastic net (1 × 1 mm mesh size), which allowed volatiles to escape.

The cylinders were divided into two equal sectors by a metal wire structure covered with a black nylon net (12 cm ID, 0.5 × 0.5 mm mesh size), which was shown to be a suitable oviposition substrate for *P. operculella* females.^13^,^14^ The odour stimuli were placed into the cage sector below the internal net, which allowed volatiles to escape in the upper sector (no-choice test). In this cage, moths could use only their olfactory ability but not their vision. Five-day-old mated females were released into the upper sector of each cage.

Synthetic compounds loaded in polyethylene dispensers were tested either alone or coupled with tubers (three tubers, overall weight 100 g cage^−1^; cv. Spunta) at doses of 40 and 4 mg. Either tubers alone or cages left empty were used as controls. Cages were kept in a climatic chamber (4 × 3 × 3 m) under controlled environmental conditions (26 ± 2 °C and 60 ± 5% RH with a 16:8 h L:D photoperiod at 2000 lux light intensity). Air in the chamber was supplied by a self-projected air flow conditioning unit equipped with filter papers, granular activated carbon filters, a laminar air flow generator and membrane humidifiers, able to modulate wind speed and relative humidity.^19^ The system provided a full air change in the chamber within 4 h. The number of eggs laid on the nylon net was counted 72 h after moths were released into the cages. Each experiment was repeated 3 times.

Parametric one-way ANOVA followed by Tukey's *post hoc* multiple comparison test was used to assess the difference in the number of eggs laid. Homogeneity of variance had been previously determined by Levene's test.

### 2.5 Warehouse experiment

The experiment was conducted in a traditional warehouse for potato storage. The volume was 18.7 m^3^ (length 2.20 m, width 3.40 m, height 2.50 m). The floor and the walls were covered with white washable tiles. Before starting the trials, the whole room was washed and ventilated for 1 week. The experiments were conducted during August 2011 at a temperature between 25 ± 1 °C and 29 ± 1 °C and at 70 ± 10% RH with a 15:9 h L:D photoperiod. The room was divided into two areas by an anti-insect net in order to allow only the odour to pass through. A quantity of 40 kg of potatoes from organic crop (susceptible varieties Spunta and Agata) were used for the trials. They were distributed in 20 crates (2 kg crate^−1^) arranged in a single layer. Half of the crates (*n* = 10) were stacked and lined up in two rows (5 crates pile^−1^, 1 pile row^−1^) spaced 1 m apart from each other, and were placed in one area of the room. The remaining ten crates were placed in the opposite area (2.5 m apart from crates in the opposite area). One octanal-loaded low-void polymer disc dispenser was placed at the centre of each crate, only in the rows of one area. No dispensers were placed in crates lined up in the opposite area. This design made it possible to create two different sets of experimental conditions in relation to the distance from the octanal source: treatment A—the dispensers baited with the tested compound were deployed directly on the potato tubers; treatment B—the potatoes were not in direct proximity to the octanal-loaded dispensers. Fifty potato tuberworm individuals (25 males and 25 females) kept in a plastic container covered with a gauze and fed with a 20% sucrose solution soaked on a cotton ball were maintained in the room for 12 h to acclimatise prior to the start of the experiment. Insects from one container were released at the centre of each of the two areas of the room, after placing the stimuli. The room was carefully sealed immediately the trial started. After 15 days of exposure, larval infestation was recorded by counting the number of potatoes with either wormholes and/or food residues. The percentage of infestation was calculated for each crate (number of damaged potatoes/total number of potatoes) and averaged for the ten replicates. At the end of each bioassay, the whole room was washed and left open for 10 days.

A control bioassay was set up in only one area of the same room, with ten crates containing potatoes left untreated (untreated control). Insects from one container were released at the centre of this area, and the percentage of infestation was calculated as described above.

Parametric one-way ANOVA followed by Tukey's *post hoc* multiple comparison test was used to assess the difference in mean tuber infestation among the two treatments and the untreated control, after checking for homogeneity of variance by Levene's test.

## 3. RESULTS

### 3.1 Single-cell recordings

#### 3.1.1 Females

Recordings from 19 sensilla trichodea showed spontaneous activity from several ORNs, which could not always be reliably distinguished by their spike amplitudes. Quantitative estimates of response strength in females are therefore based on the net responses from all neurons in the recording. Of 19 recordings with one or more responding neurons, 17 showed responses to two or more tested compounds, and the maximum number of responses from one recording was for 12 different compounds (Table1). The compounds that elicited the highest number of responses were hexanal (13 responding neurons), 1-octen-3-ol (12), octanal (10), nonanal (9), (*E,E*)-2,4-decadienal (9) and 2-hexanol (8). However, all the tested compounds elicited responses in at least two or more antennal olfactory neurons housed in trichoid sensilla. Female sensilla also contained neurons excited by (*E*,*Z*,*Z*)-4,7,10-13:Ac (5) and (*E*,*Z*)-4,7-13:Ac (3). Stimulations with aldehydes and alcohols induced the strongest increases in spike frequencies (about 50% with 70 Hz or more).

#### 3.1.2 Males

Recordings from 16 sensilla trichodea revealed at least two types of olfactory receptor neuron generating spikes of different amplitude, each of which typically responded to one of the two pheromone components (Table2). The neuron with the higher spike amplitude responded to (*E*,*Z*,*Z*)-4,7,10-13:Ac, whereas the cell with the lower spike amplitude responded to (*E*,*Z*)-4,7-13:Ac. In five cases, neurons were also excited by a host plant compound: 1-octen-3-ol twice, hexanoic acid twice and 2-hexanol once. In each case, these responses emanated from the neuron responding to (*E*,*Z*,*Z*)-4,7,10-13:Ac or possibly a third neuron with a high spike amplitude (Table2). No responses to the other plant volatiles or to the control stimuli were observed. The neuron with the higher spike amplitude showed a strong increase in spike frequency (more than 70 Hz) in 25% of the recordings, and an intermediate response (with 40–70 Hz) in 38% of the recordings after stimulation with (*E*,*Z*,*Z*)-4,7,10-13:Ac. The cells sensitive to (*E*,*Z*)-4,7-13:Ac usually gave weaker responses; at most, they fired with an intermediate rate (with 40–70 Hz) in 36% of all stimulations.

**Table 2:** Activation of receptor neurons housed in long sensilla trichodea on the antennae of male *Phthorimaea operculella* by 13 host plant volatiles and the two main pheromone components. (*E*,*Z*,*Z*)-4,7,10-13:Ac, 1-octen-3-ol, 2-hexanol and hexanoic acid evoked responses in cells with higher spike amplitudes, whereas (*E*,*Z*)-4,7-13:Ac evoked responses in cells with the lower spike amplitudes^a^

Stimuli	Recordings^b^																Number^c^
	1	2	3	4	5	6	7	8	9	10	11	12	13	14	15	16	
*Aldehydes*																	
Hexanal	0	0	0	0	0	0	0	0	0	0	0	0	0	0	0	0	0 (16)
Heptanal	0	0	0	0	0	0	0	0	0	0	0	0	0	0	0	0	0 (16)
Octanal	0	0	0	0	0	0	0	0	0	0	0	0	0	0	0	0	0 (16)
Nonanal	0	0	0	0	0	0	0	0	0	0	0	0	0	0	0	0	0 (16)
(*E*,*E*)-2,4-Decadienal	0	0	0	0	0	0	0	0	0	0	0	0	0	0	0	0	0 (16)
*Alcohols*																	
2-Hexanol	0	0	0	0	0	0	0	0	0	0	0	0	0	0	**	0	1 (16)
1-Pentanol								0	0	0	0	0	0	0	0	-	0 (8)
1-Octen-3-ol	0	0	**	0	0	0	0	0	**	0	0	0	0	0	0	0	2 (16)
*Acids*																	
Hexanoic acid	0	0	0	**	*	0	0	0	0	0	0	0	0	0	0	0	2 (16)
*Ketones*																	
2-Decanone	—	—	—	—	—	—	—	0	0	0	0	0	0	0	0	-	0 (8)
*Acetates*																	
Undecyl acetate	—	—	—	—	—	—	—	0	0	0	0	0	0	0	0	-	0 (8)
*Monoterpenes*																	
(*S*)-(−)-Limonene	—	—	—	—	—	—	—	0	0	0	0	0	0	0	0	-	0 (8)
*Sesquiterpenes*																	
(−)-Linalool	—	—	—	—	—	—	—	0	0	0	0	0	0	0	0	-	0 (8)
*Sex pheromones*																	
(*E*,*Z*,*Z*)-4,7,10-13:Ac	**	**	***	**	*	***	**	**	*	*	*	***	***	*	*	**	16 (16)
(*E*,*Z*)-4,7-13:Ac	**	**	*	**	*	*	*	*	0	0	*	**	*	*	*	**	14 (16)

a Each number in the table represents one single-cell recording.

b —: compound not tested; 0: no response by any receptor neuron

* weak response, spike frequency increase with 10–40 Hz after stimulation

** intermediate response, spike frequency increase with 40–70 Hz

*** strong response, spike frequency increase with 70 Hz or more.

c The number of recordings in which a compound elicited responses of receptor neurons in the total of recordings (in brackets).

### 3.2 Oviposition bioassay

In no-choice conditions, the number of eggs laid per cage was similar for cages containing only the suitable oviposition substrate (wire mesh) or added potato tubers as a source of volatiles. The presence of synthetic plant compounds significantly inhibited oviposition of potato tuberworm when hexanal (*F* = 717.5; df = 7, 16; *P* < 0.001), octanal (*F* = 41.4; df = 7, 16; *P* < 0.001), nonanal (*F* = 41.4; df = 7, 16; *P* < 0.001) and 1-octen-3-ol (*F* = 92.4; df = 7, 16; *P* < 0.001) were tested (Table3). Hexanal induced the highest reduction in the number of eggs laid with respect to controls. Moreover, a significant decrease in the number of eggs laid was observed with increasing hexanal dose loaded in the dispensers (Table3).

**Table 3:** Mean ± SD eggs laid by *Phthorimaea operculella* females (*n* = 3) on a cloth exposed to different odours

Stimuli^a^	Eggs laid ± SD^b^			
	Hexanal	Octanal	Nonanal	1-Octen-3-ol
4 mg	65.0 ± 8.9 b	127.3 ± 39.6 a	106.3 ± 24.8 a	72.3 ± 7.9 a
4 mg + tubers	64.3 ± 7.6 b	139.0 ± 28.4 a	121.3 ± 20.6 a	77.1 ± 8.6 a
40 mg	53.7 ± 6.0 ab	103.3 ± 14.2 a	87.3 ± 12.7 a	66.7 ± 5.2 a
40 mg + tubers	57.3 ± 4.7 ab	125.7 ± 24.0 a	105.0 ± 15.6 a	66.9 ± 7.9 a
400 mg	40.3 ± 4.2 a	100.0 ± 19.5 a	79.3 ± 16.0 a	58.3 ± 5.0 a
400 mg + tubers	46.0 ± 6.0 ab	112.7 ± 16.0 a	90.7 ± 27.8 a	60.0 ± 6.6 a
Tubers	364.0 ± 16.5 c	294.0 ± 7.0 b	271.3 ± 13.1 b	323.3 ± 42.3 b
Blank	304 ± 7.1 c	298.3 ± 14.2 b	248.0 ± 26.7 b	320.7 ± 39.5 b
ANOVA, df = 7, 16	*F* = 717.5; *P* < 0.001	*F* = 41.4; *P* < 0.001	*F* = 41.4; *P* < 0.001	*F* = 92.4; *P* < 0.001

a Doses refer to hexane solutions (100 µg µL^−1^) loaded in polyethylene dispensers applied in the cage alone or with tubers (three tubers, overall weight 100 g; cv. Spunta).

b Different letters within the same column indicate significant differences (ANOVA, Tukey's test).

### 3.3 Warehouse experiment

**Figure 1:**
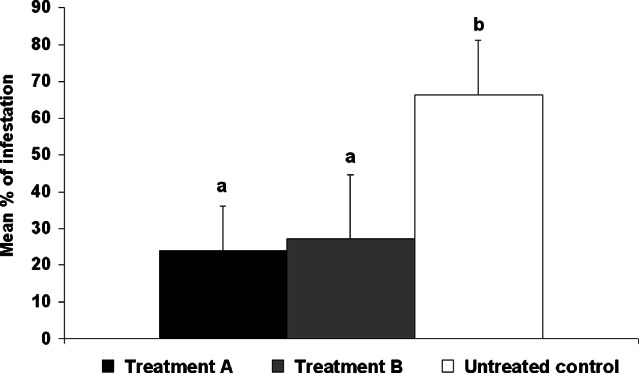
Mean percentage (±SD) of potato tubers in storage conditions damaged by *Phthorimaea operculella* treated with octanal-baited dispensers (10 mL of a 1:1 light mineral oil solution). Black bar: treatment A, octanal-baited dispensers were directly deployed on potatoes; grey bar: treatment B, potato tubers were in the same room as treatment A, 2.5 m apart from the treated side; white bar: untreated control. Different letters on the bars indicate significant differences (ANOVA, Tukey's test: *F* = 25.0; df = 2, 27; *P* < 0.001).

A significant reduction in the mean percentage of tubers damaged by *P. operculella* was observed in a comparison of treatments with octanal-baited dispensers and untreated control (*F* = 25.0; df = 2, 27; *P* < 0.001). In the ten crates in which the dispenser baited with octanal was deployed (treatment A) (Fig. 1, black bar), the mean percentage of damaged tubers was 23.9% [first-row crates (*n* = 5): 30.2%; second-row crates (*n* = 5): 17.5%]. In the ten crates of treatment B (Fig. 1, grey bar), the mean tuber damage was 27.3% [first-row crates (*n* = 5): 27.0%; second-row crates (*n* = 5): 27.6%]. In the ten control crates (Fig. 1, white bar), the mean tuber damage was 66.3% [first-row crates (*n* = 5): 60.5%; second-row crates (*n* = 5): 72.1%]. The reduction is statistically relevant either when octanal-baited dispensers were directly deployed on potatoes (treatment A) (Fig. 1, black bar) or when tubers were in the opposite area of the room 2.5 m apart from the octanal dispensers (treatment B) (Fig. 1, grey bar). No significant differences were observed between treatments A and B (*F* = 25.0; df = 2, 27; *P* < 0.001).

## 4. Discussion

In potato tuberworm females, long sensilla trichodea apparently function as broad-spectrum sensors for a great number of odours ranging from plant volatiles to pheromone compounds. The authors could not with certainty distinguish responses of single olfactory cells, and therefore were unable to determine the degree of specificity of individual receptor neurons. However, the ability of females to detect their own sex pheromone has already been demonstrated with both electrophysiological and behavioural experiments in several species of Lepidoptera, i.e. *Trichoplusia ni* (Hübner),^20^ *Choristoneura fumiferana* (Clemens),^21^ *Adoxophyes orana* (Fischer von Röslerstamm),^22^ *Spodoptera littoralis* (Boisduval) and *Spodoptera frugiperda* (Smith),^23^,^24^ *Euplagia* (*Panaxia*) *quadripunctaria* (Poda),^25^ *Cydia splendana* (Hübner) and *Cydia fagiglandana* (Zeller)^26^ and *Cydia pomonella* (L.),^27^,^28^ and it has been proposed that this perception could play a role in the optimisation of pheromone production and in the spatial dispersion of calling females over the host plants.^21^,^22^,^29^,^30^

Long sensilla trichodea located on the surface of male antennae mainly function as specific pheromone receptors. They house at least two different olfactory cells specialised to detect only one of the components of the female sex pheromone, as reported for other lepidopteran species.^26^,^31^,^32^ It is worth noting that these specialised male receptor neurons occasionally but selectively respond to host plant compounds with chemical structures not related to those of pheromones, or that other neurons with different response specificities are possibly sometimes also present in these sensilla. Moreover, it cannot be ruled out that spikes from neighbouring sensilla have also been recorded.^33^ The possible existence of either sensilla or receptor cells responding both to plant volatiles and pheromone components might supply further interpretations concerning the reported ability of plant compounds to modulate the behavioural responses to pheromones in insect species.^27^ Host plant volatiles can indeed synergise the attractant power of a synthetic sex pheromone but can also have inhibitory or repellent effects that disrupt insect responses to pheromones.^34^–^42^ Therefore, these interactions in odour perception at a peripheral level need to be carefully considered when setting up new semiochemically based monitoring or control methods.

In the no-choice behavioural bioassay the four most SCR-active volatiles from potato, hexanal, octanal, nonanal and 1-octen-3-ol strongly disrupted the oviposition site selection process by potato tuberworm females when individually applied at concentrations considerably above their production in intact and healthy plant tissues. This negative effect of unbalanced amounts of individual host plant volatiles on moth's egg-laying behaviour is consistent with the observation that in polyphagous insects the absolute and relative amounts of ubiquitous plant volatiles are critical factors in host selection.^43^–^46^ Moreover, such a disruptive effect may be due to the insect's ability to avoid a source of toxic compounds. It is well known that the emission of green-leaf volatiles, including short-chained aliphatic aldehydes, alcohols and esters, dramatically increases when the plant tissues are wounded.^47^ These compounds are produced through the hydroperoxide lyase pathway of oxylipin metabolism^48^ in response to mechanical and herbivory damage and can play a major role in plant defence. For example, aliphatic aldehydes are known to possess fumigant and contact^49^,^50^ and repellent effects,^51^,^52^ depending on the dose, against various insect pests, including potato tuberworm females in laboratory oviposition bioassays.^11^ Moreover, some of them were found to inhibit pathogenic fungi^53^,^54^ and bacteria.^55^ Interestingly, synthetic plant volatiles, either corresponding (octanal and nonanal) or chemically related, (*Z*)-3-hexenol and (*E*)-2-hexenal, to those selected in the present study, were also shown to attract beneficial insects when deployed at high dosages in different agroecosystems.^56^,^57^ Moreover, potato tuberworm females are attracted to volatiles released by intact potato tubers but not to those from tubers damaged by conspecific larvae, which, in turn, orientate the natural enemy *Orius insidiosus* (Say).^12^

In no-choice conditions, the olfactory cues from tubers were not able to enhance potato tuberworm oviposition in comparison with a control represented by an adequate physical substrate for oviposition, as demonstrated in previous studies,^13^,^14^ whereas the same odour has been shown to mediate female attraction to the plant at longer range.^11^,^12^ This suggests that potato tuberworm females probably use different cues for host plant recognition and oviposition site location respectively.

The experiments conducted under conventional storage conditions confirmed the results of laboratory assays. Octanal was preferred to hexanal because, even though slightly less effective, it has a lower volatility (octanal vapour pressure 1.2 mmHg at 20–25 °C, hexanal vapour pressure 11 mmHg at 20–25 °C)^58^ and hence is more suitable for a longer release duration in practical applications. In the warehouse, octanal elicited a strong decrease in the number of eggs laid compared with the untreated control, both when dispensers were directly deployed on potatoes and when tubers were 2.5 m apart from the treated side, probably as a result of a homogeneous dispersion of the compound in the surroundings. The present results therefore suggest the possibility of avoiding the direct contact of octanal with stored potatoes, and even of using a relatively low density of semiochemical-baited dispensers. In South-East Asia, repellent plant materials applied as dried leaves or powders just to cover stored-tuber bulks are reported to be an effective control strategy to prevent *P. operculella* infestation.^59^–^61^

In conclusion, this work has provided new information on the olfactory system of potato tuberworm, with particular attention to the perception of intra- and interspecific semiochemicals by long sensilla trichodea in both sexes. Moreover, laboratory and semi-field behavioural studies have shown that, among alternative botanical control means,^59^,^62^ the use of synthetic plant volatiles, i.e. common short- and straight-chained aldehydes and alcohols, as host recognition disruptant and/or oviposition deterrent for potato tuberworm appears to be feasible under potato storage conditions.

Further studies are now required to define large-scale application methods utilising these bioactive compounds against potato tuberworm.
